# Exome Sequencing in Individuals with Isolated Biliary Atresia

**DOI:** 10.1038/s41598-020-59379-4

**Published:** 2020-02-17

**Authors:** Ramakrishnan Rajagopalan, Ellen A. Tsai, Christopher M. Grochowski, Susan M. Kelly, Kathleen M. Loomes, Nancy B. Spinner, Marcella Devoto

**Affiliations:** 1 Division of Genomic Diagnostics, Department of Pathology and Laboratory Medicine, Children’s Hospital of Philadelphia, Philadelphia, PA USA; 2 Genomics and Computational Biology Graduate Group, The University of Pennsylvania, Philadelphia, PA USA; 3 Genetic Epidemiology Group, Department of Translational Biology, Biogen, Cambridge, MA USA; 4 Department of Molecular and Human Genetics, Baylor College of Medicine, Houston, TX USA; 5 Division of Gastroenterology, Hepatology and Nutrition, Department of Pediatrics, Ann and Robert H. Lurie Children’s Hospital of Chicago, Chicago, IL USA; 6 Division of Gastroenterology, Hepatology and Nutrition, Children’s Hospital of Philadelphia, Philadelphia, PA USA; 7 Department of Pediatrics, Perelman School of Medicine, University of Pennsylvania, Philadelphia, PA USA; 8 Department of Pathology and Laboratory Medicine, Perelman School of Medicine, University of Pennsylvania, Philadelphia, PA USA; 9 Division of Human Genetics, Children’s Hospital of Philadelphia, Philadelphia, PA USA; 10 Department of Translational and Precision Medicine, University La Sapienza, Rome, Italy

**Keywords:** Rare variants, Cholestasis

## Abstract

Biliary atresia (BA) is a severe pediatric liver disease resulting in necroinflammatory obliteration of the extrahepatic biliary tree. BA presents within the first few months of life as either an isolated finding or with additional syndromic features. The etiology of isolated BA is unknown, with evidence for infectious, environmental, and genetic risk factors described. However, to date, there are no definitive causal genes identified for isolated BA in humans, and the question of whether single gene defects play a major role remains open. We performed exome-sequencing in 101 North American patients of European descent with isolated BA (including 30 parent-child trios) and considered several experimental designs to identify potentially deleterious protein-altering variants that may be involved in the disease. In a case-only analysis, we did not identify genes with variants shared among more than two probands, and burden tests of rare variants using a case-case control design did not yield significant results. In the trio analysis of 30 simplex families (patient and parent trios), we identified 66 *de novo* variants in 66 genes including potentially deleterious variants in *STIP1* and *REV1*. STIP1 is a co-chaperone for the heat-shock protein, HSP90, and has been shown to have diverse functions in yeast, flies and mammals, including stress-responses. REV1 is known to be a key player in DNA repair pathway and to interact with HSP90. In conclusion, our results do not support the hypothesis that a simple genetic model is responsible for the majority of cases of isolated BA. Our finding of *de novo* variants in genes linked to evolutionarily conserved stress responses (*STIP1* and *REV1*) suggests that exploration of how genetic susceptibility and environmental exposure may interact to cause BA is warranted.

## Introduction

Biliary atresia (BA) is an obstructive cholangiopathy with initial symptoms arising during the first days to weeks of life. BA occurs as an isolated finding in 85% of affected individuals, and with additional syndromic features (heterotaxy and/or other congenital birth defects) in 15%^1^. The incidence of BA varies across different populations, with estimates ranging from 1 in 5,000 to 1 in 14,000 live births^2^. BA presents clinically with neonatal cholestasis, elevated bilirubin and liver enzymes, although the differential diagnosis suggested by these findings is extensive. The diagnosis of BA is suggested by features of biliary obstruction on liver histology, including bile duct proliferation, portal tract expansion and bile plugs. The diagnosis is confirmed upon intraoperative cholangiogram showing lack of patency of the extrahepatic biliary tree.

Although the etiology of BA is not clear, a genetic component is supported by multiple lines of evidence. These include the presence of familial cases^3–5^, case reports of individuals with syndromic BA with likely causal genes identified (including *FOXA2*^6^, *CFC1*^7,8^, *ZEB2*^9^, *ZIC3*^10^, *HNF1B*^11^ and *PKD1L1*^12^), animal models of BA involving the genes *Sox17*^13^, *Foxm1*^14^, *Invs*^15^, *Onecut1*^16^, and candidate susceptibility genes including *ADD3*^17^, *GPC1*^18^, *EFEMP1*^19^ and *ARF6*^20^ identified by genome-wide association studies (GWAS). A follow-up study of *GPC1* using a zebrafish model confirmed that suppressing *GPC1* expression gives rise to biliary defects^21^. A GWAS identified SNPs associated with BA upstream of *XPNPEP1* and *ADD3* in a Chinese cohort of isolated BA patients^17^, and this association has been confirmed in both a Thai^22^ and a European-American cohort^23^. In a study of 499 isolated and syndromic BA patients and 1928 controls, our group identified a GWAS signal within 2p16.1 implicating common variants in the gene *EFEMP1*^19^. Another genome-wide association study of 63 BA patients and 1907 controls identified two SNPs downstream of the gene *ARF6* associated with isolated BA^20^, although we could not replicate this finding in our study. However, to date, no genes have been definitively identified as a cause of isolated BA. Environmental exposure to viruses or toxins has long been proposed as a hypothesis to explain the etiology of the perinatal form of BA^2^, but the genetic susceptibility factors linking such exposures and BA are yet to be identified.

In this work, we hypothesized that rare and novel, deleterious, protein-altering variants transmitted in a Mendelian fashion could be involved in the etiology of isolated BA, and patients with isolated BA would be enriched with such variants. To test this hypothesis, we considered three experimental designs (Table 1). First, we conducted a case-only analysis where we looked for potentially deleterious novel variants. In the second design, we conducted a case-control analysis comparing the cumulative frequency of rare variants in the two groups. Finally, we performed trio analysis on a subset of 30 probands to identify genes with rare, protein-altering *de novo* and compound heterozygous or homozygous variants.

**Table 1:** Experimental designs and variant prioritization schemes used in this study.

Experimental design	Case-only	Case-control	Trio	
			*de novo*	Recessive
Subjects	100 cases	100 cases, 303 controls	30 parent-child trios	30 parent-child trios
CADD score threshold	≥30	none	none	≥30
AF threshold in gnomAD v2.1 NFE	0	none	none	≤0.1%
AF threshold in cohort	none	<5%	none	none
Statistical test	Over-representation	Burden	NA	NA

Variant types include stop gain, stop loss, frameshift and in-frame indels, missense, and splicing (splicing variants include both canonical sites (+/−1–2 bp) as well as 3–8 bp into the introns). *Abbreviations: CADD – Combined Annotation-Dependent Depletion score; AF- Allele Frequency; gnomAD – Genome Aggregation Database; NFE- Non-Finnish Europeans*.

## Patients and Methods

### Patients with isolated BA and controls

We studied a cohort of 101 self-reported white, non-Hispanic patients with isolated BA. The patients were ascertained from a larger cohort of 1063 BA cases based on their self-reported race and ethnicity, availability of parental DNA samples, and absence of any other anomaly. Ninety-nine participants were enrolled in the NIDDK-funded Childhood Liver Disease Research Network (ChiLDReN) at one of the 16 participating institutions under Institutional Review Board (IRB) approved protocols (IRB 04–003655 and 06–004814). A set of affected dizygotic twins were consented into the approved IRB study at the Children’s Memorial Hospital, Chicago, IL. One of them was randomly selected for inclusion in the cohort of unrelated individuals (for over-representation and burden tests). Informed consent was obtained from parents or legal guardians for each patient enrolled in this study. The diagnosis of BA was made by clinical presentation, liver histology, and an intraoperative cholangiogram. Most patients also had BA confirmed by examination of the biliary remnant from a Roux-en-Y hepatic portoenterostomy (also known as a Kasai operation). The average age at Kasai was 0.16 years (range 0–1.16 years). There were no data available on BA subtype and gestational age for the majority of individuals included in the study. DNA samples were extracted from whole blood (n = 16) or lymphoblastoid cell lines (LCLs) (n = 85). For a subset of patients (n = 32), DNA samples were also available from both parents (LCL = 56; whole blood = 6).

Controls were individuals recruited from two different studies at the Children’s Hospital of Philadelphia, PediSeq (the CHOP cohort of the Clinical Sequencing and Exploratory Research (CSER) consortium)^24^, and Very Early Onset Inflammatory Bowel Disease (VEO-IBD)^25^, and enrolled in institution-approved IRB protocols (IRB 12–009169; 14–010826). Controls from the PediSeq dataset (n = 158) fall into four different disease cohorts (cardiovascular, hearing loss, mitochondrial and intellectual disability) but have no known liver disease. Controls from the VEO-IBD cohort (n = 145) were the unaffected parents of individuals with VEO-IBD. The patient recruitment, methods, and experimental protocols used in these studies were performed in accordance with relevant guidelines and regulations set by the aforementioned institutions. This study was approved by the Ethics Committee of the Children’s Hospital of Philadelphia.

### Exome sequencing

Genomic DNA (3–5 ug) was supplied to Perkin Elmer, Inc. (Perkin-Elmer, Branford, CT) for exome sequencing of the BA patients and their parents. The DNA was amplified and enriched using the Agilent SureSelect V4 + UTR 71 Mb All Exon Capture Kit (Agilent Technologies, Santa Clara, CA). Sequencing was performed on the Illumina HiSeq 2000 (Illumina, San Diego, CA) at the Perkin-Elmer sequencing service center. Control samples were sequenced using Agilent SureSelect V4 51 Mb Exon Capture Kit (Agilent Technologies, Santa Clara, CA) on the Illumina HiSeq 2000 (Illumina, San Diego, CA) at the internal sequencing core at CHOP (BGI@CHOP, Philadelphia, PA). All of the exome sequencing data was produced with paired-end reads of 100 base-pairs in length (2 × 100 bp). The unaligned FASTQ reads of all samples were returned to us for read analysis and downstream processing as detailed below.

### Variant calling and annotation

Raw sequencing reads were mapped to the GRCh37.69 reference genome using the Burrows-Wheeler Aligner^26^ to produce an aligned BAM file. BAM files were realigned around known indels using IndelRealigner in the GenomeAnalysisToolKit software (GATK) and base quality scores were recalibrated using the BaseQualityScoreRecalibrator in GATK^27^. Variant calling was performed using HaplotypeCaller and jointly genotyped using the GenotypeGVCFs command in GATK. The initial variant quality filtering was done using the VariantQualityScoreRecalibrator (VQSR) in GATK. SNPEff^28^ was used to annotate the variants with their expected functional consequence. Other annotations such as allele frequencies in non-Finnish Europeans (NFE) from the gnomAD variant database version 2.1^29^, and deleteriousness scores such as CADD (Combined Annotation Dependent Depletion version 1.4)^30^ were added using the Gemini software^31^. We manually reviewed all variants found in the *de novo* analysis, and variant clusters with overlapping genomic positions that could be indicative of technical artifacts^32^. Individual genes were further annotated using the pLI, or probability of loss-of-function intolerance, a gene-level measure estimated by comparing the expected number of loss-of-function variants against the number observed in a population. The pLI score ranges from 0 to 1 with the highest value being the most intolerant^33^.

### Quality control

The Peddy^34^ software tool was used to infer genetic ancestry from the exome sequencing data by comparing our samples against the samples from the 1000 Genomes Project Phase 3^35^. All samples in the case and control cohorts clustered with the European populations in the 1000 Genomes Project confirming their self-reported race/ethnicity (Supp. Fig. 1). We also confirmed the sex of the participants based on heterozygosity of X chromosome variants. Pairwise relatedness (estimated by the kinship coefficient) between samples was investigated using the KING^36^ software, and confirmed that there were no related individuals in the entire cohort beside the known parent-child pairs used in the trio analysis and the twins. Distribution of the variant allele fraction (allele balance) for all heterozygous variants identified in the cases (Supp. Fig. 2), principal component analysis of pairwise genetic distances based on genotype data (Supp. Fig. 3), and the distribution of transition/transversions ratio (Supp. Fig. 4) stratified by source of DNA were used to compare results obtained from samples sequenced from whole-blood and LCLs, and did not reveal any systematic differences between the two sets of samples.

### Variant prioritization

We filtered the variants identified from exome sequencing for stop gain, stop loss, frameshift and in-frame insertions/deletions (indels), missense and splicing variants. We excluded variants overlapping with segmental duplications and variants with call rates less than 90% from all analyses. In the case-only analysis, we included novel and potentially deleterious variants, defined as variants that were not present in the NFE population of the gnomAD v2.1 genomes and exomes and had a phred-scaled CADD score of at least 30, representing the top 0.1% of the potentially deterious variants. For the burden tests in the case-control experimental design, all protein-altering variants with a cohort (cases and controls combined) minor allele frequency (MAF) of <5% were included. We did not apply a CADD score threshold for this analysis. For the *de novo* analysis, we considered all variants irrespective of their frequency or the CADD score. For the analysis under the recessive model, we retained variants with an allele frequency in gnomAD v2.1 NFE less than or equal to 0.1% and a CADD score of at least 30. Details pertaining to variant prioritization are provided in Table 1.

### Autosomal copy-number variant (CNV) analysis

Mean depth of coverage for each individual exonic interval in the autosomes was computed using the DepthOfCoverage routine in the GATK package and copy number variants were called using XHMM following the protocol described elsewhere^37^. We excluded CNVs with quality score (Q_SOME) less than 60 to retain only high quality CNVs. To identify novel CNVs, we excluded any variant found in the Database of Genomic Variants (DGV)^38^ and the ExAC version 1.0^39^. We did not look for CNVs in the sex chromosomes as the ExAC dataset only had calls from autosomes.

### Gene, pathway and gene-set definitions

For all the analyses, we used refFlat_hg19 gene definitions from the UCSC genome browser^40^. We used pathway definitions including KEGG (n = 293), Panther (n = 112), Reactome (n = 1530), WikiPathways (n = 437) and BioCarta (n = 237), and annotation gene-sets from Gene Ontology (GO) Biological Process (n = 5192), Molecular Functions (n = 1136) and Cellular Components (n = 641) downloaded from the Enrichr website^41^.

### Case-only and trio analyses: Over representation tests

To determine if our lists of candidate genes from the case-only and trio analyses were enriched for specific gene-sets or pathways, we performed over-representation analysis using the R package enrichR^41^. This software essentially counts the number of genes with a given annotation that are present in our lists of candidate genes and genome-wide, and compares it to the number of genes that are not associated with the same annotation in our lists and genome-wide, using a Fisher exact test. Over representation tests were carried out on gene-sets and pathways.

### Case-control analysis: burden tests

To test if a gene, gene-set or pathway contained a significantly different number of low frequency or rare variant (cohort MAF < 5%) in cases compared to controls, we performed two-sided Fisher’s exact tests using the Combined and Multivariate Collapsing (CMC) approach^42^ implemented in the exactCMC procedure from the software package rvtests^43^. Burden tests were carried out at different levels including (1) genes; (2) gene-sets (e.g. collection of genes with similar function or structure or cellular localization, such as sodium channel genes or membrane bound receptors); and (3) pathways, which include genes that function in a specific biological pathway (e.g. WNT or NOTCH signaling). The number of cases and controls with and without at least one rare variant in a given gene, gene-set, or pathway were compared using a two-sided Fisher exact test.

### Trio and quad analysis

Segregation analysis was performed using Gemini software^31^ in a subset of 30 probands and a dizygotic twin pair for whom DNA from both parents was available. *De novo* variants were identified using the command ‘*de_novo*’ which detects SNVs and indels occuring in the proband and not in the parents. Compound heterozygous variants were identified using the command ‘*comp_het*’ which detects two heterozygous variants in the same gene inherited from different parents. Homozygous variants were identified using the ‘*autosomal_recessive*’ command which detects variants present in homozygosity in the proband and in heterozygosity in both parents.

### Statistical significance

For gene level burden tests, the raw p-values were corrected for multiple testing using a Bonferroni correction for the total number of genes tested (n = 16,393). For the pathway based analyses, the raw P-values were corrected for multiple testing using a Bonferroni correction for the total number of pathway definitions (n = 2609). For the gene-set based analyses, we corrected for the total number of gene-sets used in the analysis (n = 6969). We considered a test to be statistically significant if the Bonferroni-adjusted p-value was less than or equal to 0.05. Post-hoc power analysis for the case-control burden analysis was performed using the software tool G*Power^44^. We used the maximum observed effect size from the gene-level burden test to compute the minimum sample size required to observe the same difference at a Bonferroni-corrected significance level based on the number of genes tested (p < 3 × 10^−6^).

## Results

### Case-only analysis

We identified a total of 496,654 variants that passed the quality filters in the 100 probands. Out of 63,473 single nucleotide variants (SNV) or small indels found to be protein-altering changes (stop gain, stop loss, frameshift and in-frame indels, missense and splicing variants), 332 variants in 326 genes were novel (not in the NFE population in gnomAD exomes and genomes) and had a phred-scaled CADD score of 30 or more. This formed our variant dataset for the case-only analyses. Almost all of the variants (331/332) were present in heterozygosity in a single individual, and 96 out of the 100 individuals in the cohort had at least one potentially deleterious novel variant. Six genes had two different variants in two different individuals *(MAST2, WDR35, DNAH5, FBN2, SCUBE2* and *PITPNM3)*. Over-representation analysis did not yield any significant gene, pathway or gene-set after correcting for multiple testing. Copy-number variant analysis did not reveal any novel variant in the probands after filtering for variants present in the population databases ExAC and DGV. Details of the SNVs and indels with annotations are provided in the Supp. Table 1.

### Case-control analysis

In the second experimental design, we conducted burden tests for low frequency and rare variants (MAF < 5%) by comparing their cumulative number in a gene, gene-set or pathway in the 100 cases against the 303 controls. After correcting for multiple testing, we did not identify any gene, gene-set or a pathway with a statistically significant difference in burden of low frequency and rare variants in the cases compared to the controls. Analysis of QQ-plot from the gene-based burden tests of genes with three or more variants did not show a deviation of the observed p-values compared to the expected distribution (Supp. Fig. 5). Post-hoc power analysis using the most significant result from the gene-based burden analysis (proportion of individuals with a rare variant in cases = 2% and controls = 15%; p-value = 0.0001) suggested that 260 cases and controls (total n = 520) are required to achieve 80% power for the same effect size to reach statistical significance after correction for the number of genes tested (p < 3 × 10^−6^).

### Trio and quad analysis

In the final experimental design, we sequenced the parents of a subset of the patients (30 singletons and one affected dizygotic twin pair) to identify *de novo* variants in the probands under the hypothesis that they are more likely to be involved in the etiology of the disease, and variants transmitted from the parents to the probands according to a recessive model (homozygous or compound heterozygous). We identified a total of 66 *de novo*, protein-altering and splice variants in 66 different genes in 25 out of 30 individuals, with a mean of 2.2 variants per individual (range: 0–7; median 3). We did not identify any *de novo* variants in five individuals. All the *de novo* variants were manually verified in the BAM file and a small subset of them were validated using Sanger sequencing (n = 4). There were 58 missense, 3 frameshift indels, 3 nonsense, and 2 splice-site variants. A prioritized list of 14 *de novo* variants (all loss-of-function variants, and missense variants with CADD greater than or equal to 30) is provided in Table 2, and all *de novo* variants are listed in the *Supp*. Table 2. Of the eight *de novo* loss-of-function variants, a premature stop-gain in the gene *STIP1* (NM_001282652: p.(Cys30Ter)) and a splice-acceptor variant in the gene *REV1* (NM_001037872: c.1674-2 A > G) were the most interesting as these genes are known to be intolerant to loss-of-function, with a pLI (probability of loss-of-function intolerance) score of 1. Sanger traces and a graphical representation of the sequence reads from the BAM files supporting their presence (read pile-up), are provided for these two variants in Supp. Figs. 6 and 7. Over-representation analysis of the 66 genes did not yield significant results after correcting for multiple testing.

**Table 2:** Prioritized list of *de novo* variants identified in the analysis of 30 parent-child trios.

Chr	Position	Ref	Alt	dbSNP ID	gnomAD v2.1 AF	Gene symbol	Impact	Amino acid change
	**All** ***de novo*** **loss-of-function variants**							
2	100038117	T	C		0	*REV1*	splice_acceptor	NM_001037872:exon11:c.1674-2 A > G NM_016316:exon11:c.1677-2 A > G
3	47048735	CTG	C		0	*NBEAL2*	frameshift	NM_015175:exon47:c.7230_7231del:p.T2410fs
8	145621814	CCT	C	rs782640869	0	*CPSF1*	frameshift	NM_013291:exon25:c.2823_2824del:p.S941fs
9	114804380	C	A	rs781608602	8.3 × 10^−5^	*SUSD1*	splice_donor	NM_001282640:exon17:c.2214 + 1 G > T
11	63953386	C	A	rs545010478	2.2 × 10^−5^	*STIP1*	stop_gained	NM_001282652:exon1:c.C90A:p.C30X
17	34165489	C	CTT		0	*TAF15*	frameshift	NM_003487:exon11:c.836_837insTT:p.P279fs NM_139215:exon11:c.845_846insTT:p.P282fs
20	16360632	A	T		0	*KIF16B*	stop_gained	NM_001199865:exon19:c.T2015A:p.L672X NM_001199866:exon19:c.T2015A:p.L672X NM_024704:exon19:c.T2015A:p.L672X
X	140969336	C	A		0	*MAGEC3*	stop_gained	NM_138702:exon4:c.C663A:p.Y221X
	**Non-synonymous** ***de novo*** **variants with CADD ≥ 30**							
2	63834044	C	T		0	*MDH1*	missense	NM_001199112:exon8:c.C661T:p.R221C NM_001199111:exon9:c.C982T:p.R328C NM_005917:exon9:c.C928T:p.R310C
2	68873329	C	T	rs771784209	2.5 × 10^−5^	*PROKR1*	missense	NM_138964:exon1:c.C376T:p.R126C
4	158142842	G	T		0	*GRIA2*	missense	NM_000826:exon2:c.G112T:p.D38Y NM_001083619:exon2:c.G112T:p.D38Y
5	52780025	G	A		0	*FST*	missense	NM_006350:exon4:c.G623A:p.G208E NM_013409:exon4:c.G623A:p.G208E
11	4095877	C	T		0	*STIM1*	missense	NM_001277961:exon7:c.C937T:p.R313C NM_001277962:exon7:c.C937T:p.R313C NM_003156:exon7:c.C937T:p.R313C
17	38319096	G	A		0	*CASC3*	missense	NM_007359:exon6:c.G727A:p.D243N

This table lists all the loss-of-function and splice variants, and missense variants with a CADD score greater than or equal to 30. *Abbreviations: dbSNP – Database of Single Nucleotide Polymorphisms; CADD – Combined Annotation-Dependent Depletion score*.

By segregation analysis, we did not identify any compound heterozygous or homozygous variants at a maximum alternate allele frequency threshold of 0.1% and a phred-scaled CADD score of 30 in the 30 trios. After applying the same filters, we did not identify *de novo*, compound heterozygous or homozygous variants shared by both affected individuals in the twin quad.

## Discussion

In this study, exome sequencing was used to identify genomic changes in a cohort of patients with isolated biliary atresia, and in spite of the utilization of several distinct strategies, we were unable to demonstrate an associated genetic change. We did identify new candidate genes by analysis of 30 trios, finding potentially deleterious *de novo* variants in *STIP1* and *REV1*, which we hypothesize may result in susceptibility to toxin induced biliary disease as discussed below.

BA presents as an isolated finding in 85% of cases, and in a syndromic form with laterality and/or other congenital malformations in 15%. Mouse models of BA (*Sox17, Onecut1*) and gene mutations in patients with syndromic forms of BA (*CFC1, ZIC3, FOXA2, PKD1L1*) suggest an etiology involving genetic factors that influence left-right symmetry, and bile duct development and morphology. Genome-wide association studies in patients with isolated BA have suggested candidate genes and genomic regions (*ADD3* and *XPNPEP1* in 10q25.1, *EFEMP1* in 2p16.1, *GPC1* in 2q37.3, *ARF6* in 14q21.3) but consistent proof of the involvement of each of these regions has been elusive. BA as a consequence of exposure to environmental toxins is also proposed as a model for the perinatal form of the disease based on research into the natural occurance of BA in Australian sheep, which led to the discovery of an isoflavonoid (biliatresone) found in specific plant species, later found to cause biliary defects in zebrafish^45^. RNA-seq experiments on biliatresone treated and control fish identified several differentially expressed stress signaling pathways including the glutathione antioxidant pathway, cellular redox response, and heat shock response^46^. However, the genetic susceptibility factors that might link exposure to these agents and BA are yet to be identified. In summary, from previous literature, there are genes that are known to cause syndromic forms of BA in humans and mice, genes that are associated with isolated BA identified via GWAS, and biological pathways that are perturbed in zebrafish treated with the toxin biliatresone, which has been associated with BA in sheep. However, in spite of these clues, there has not been a causal gene identified for isolated BA in humans.

In this work, we performed exome sequencing in 101 North American patients of European ancestry with isolated BA and looked for novel or low-frequency and rare protein-altering variants that may explain the BA phenotype (strategies presented in Table 1). Such study designs have been successful in finding novel genes responsible for rare Mendelian diseases^47^. Fewer exome sequencing studies with sufficiently large sample sizes have been successfully employed in identifying associations with complex phenotypes^48,49^. This study was primarily designed with the goal of finding genes with rare and highly deleterious variants that may explain the occurrence of isolated BA. We considered three experimental designs including a case-only analysis to look for novel, potentially deleterious and protein-altering variants, a case-control analysis to look for an excessive burden of low frequency and rare variants in cases compared to controls, and a segregation analysis, to look for *de novo* and homozygous or compound heterozygous variants.

In the case-only design, we identified 332 novel and potentially deleterious protein-altering variants in our cohort of 100 unrelated individuals with isolated BA. The majority of the variants were found in heterozygosity in a single individual. Six genes had variants in two individuals, and these variants were different from each other. This suggests that BA is unlikely to result from a single genetic change, although we cannot rule out extreme locus heterogeneity, with a small percentage of cases having a genetic etiology, perhaps in combination with an environmental insult. Case-control analysis did not yield any statistically significant result, and this may suggest that there are no variants or genes with large enough effect sizes present in the cases as compared to the controls or our sample size is too small to identify such a difference between cases and controls.

Trio-based segregation analysis identified 66 *de nov*o potentially protein-altering variants in 66 genes in 25 out of 30 unrelated patients. All putative loss-of-function *de novo* variants and a prioritized list of *de novo* missense variants with a CADD score greater than or equal to 30 are included in Table 2. Of all the genes with putative loss-of-function *de novo* variants, a splice-acceptor variant in the gene *REV1* and a premature stop in one of the transcripts in the gene *STIP1* were the most interesting for several reasons. These genes are known to be intolerant to loss-of-function variants with a perfect pLI score of 1. STIP1 is a chaperone that assists in the transfer of proteins from HSP70 to HSP90, which are an integral part of the heat shock response pathway. Mass spectrometry experiments to identify differentially expressed proteins in 20 BA liver biopsies compared to 12 non-BA, neonatal cholestasis livers found that HSP90 was significantly down-regulated in BA livers, and was the most significantly altered protein^50^. *REV1* encodes a protein similar to the *S. cerevisiae* mutagenesis protein Rev1 and is known to be involved in DNA repair. REV1 is shown to be regulated by the heat-shock protein HSP90 in the DNA repair pathway^51^. Based on the *de novo* variant found in the human exome data, along with evidence that the heatshock response pathway is differentially expressed in zebrafish treated with the plant toxin biliatresone, our collaborators used CRISPR/Cas9 methods to introduce a frameshift mutation in the zebrafish *stip1* gene. When treated with biliatresone toxin, *stip1* heterozygous fish were highly sensitive to a low dose that is normally inactive in wild-type fish^52^. Similar sensitization was detected in human cholangiocytes following siRNA knockdown of the *STIP1* isoform targeted by the BA variant^52^. Heterozygous *rev1* mutation in zebrafish and siRNA knockdown of *REV1* in human cholangiocytes were sensitized to low dose biliatresone, similar to mutation and knockdown of *stip1/STIP1*^52^.

Our study has several limitations including a small sample size (which is a function of the disease frequency) and the failure of exome sequencing to identify all possible disease causing genomic changes. The sample size was relatively small for statistical analyses such as rare variant burden tests and for the trio analysis to identify genes with recurring *de novo* variants. Exome sequencing is a targeted capture experiment which focuses only on the exonic regions^53^, and coverage might not be uniform through the coding parts of the genome. This limits our ability to study intronic regions and/or poorly targeted parts of the genome. Copy-number detection from exome sequencing is challenging and the XHMM software tool used in this work is tuned for specificity rather than sensitivity. In addition, exome sequencing does not allow us to look for structural variation in these samples. Finally, the majority (85/101) of the DNA samples used in this study came from LCLs, including those containing the *de novo* variants in *STIP1* and *REV1* discussed above, and we do not have access to a primary source of DNA to confirm their presence. It is known that LCLs are susceptible to mutational changes over passages (cell line artifacts)^53^. However, in our study, we did not see any evidence of systematic differences in the results obtained from DNA samples extracted from whole blood or LCLs.

Together, our analyses do not support a simple genetic model in which a small number of genes are responsible for the majority of cases with isolated BA. However, the identification of *de novo* variants in the genes *STIP1* and *REV1* and the evidence for their sensitivity to the biliatresone toxin in zebrafish open new opportunities for further investigating BA as a consequence of environmental exposure in genetically susceptible individuals. Other genes in the heat shock response pathway or other pathways identified in the zebrafish experiments could also be considered as BA susceptibility candidate genes.

## Supplementary information

Supplementary Figures 1 to 7.

Supplementary Tables 1 and 2.
