# Supplementary material for: Exome Sequencing in Individuals with Isolated Biliary Atresia
Source: Sci Rep. 2020 Feb 17;10:2709. doi: 10.1038/s41598-020-59379-4 (PMC7026070; doi:10.1038/s41598-020-59379-4)
Supplement: Supplementary file 1 — Supplementary Figures 1 to 7. [file 41598_2020_59379_MOESM1_ESM.pdf]

Exome Sequencing in Individuals with Isolated Biliary Atresia

Ramakrishnan Rajagopalan[1], Ellen A. Tsai[1,2,3], Christopher M. Grochowski[1,4], Susan M. Kelly[5], Kathleen M. Loomes[6,7], Nancy B. Spinner[1,8], Marcella Devoto[7,9,10]*

[1]Division of Genomic Diagnostics, Dept. of Pathology and Laboratory Medicine, Children's Hospital of Philadelphia, Philadelphia, PA, USA.

[2]Genomics and Computational Biology Graduate Group, The University of Pennsylvania, Philadelphia PA, USA.

[3]Genetic Epidemiology Group, Dept. of Translational Biology, Biogen, Cambridge, MA USA.

[4]Department of Molecular and Human Genetics, Baylor College of Medicine, Houston, TX, USA.

[5]Division of Gastroenterology, Hepatology and Nutrition, Department of Pediatrics, Ann and Robert H. Lurie Children's Hospital of Chicago, Chicago, IL, USA.

[6]Division of Gastroenterology, Hepatology and Nutrition, Children's Hospital of Philadelphia, Philadelphia, PA, USA.

[7]Department of Pediatrics, Perelman School of Medicine, University of Pennsylvania, Philadelphia, PA, USA.

[8]Dept. of Pathology and Laboratory Medicine, The Perelman School of Medicine at The University of Pennsylvania, Philadelphia PA.

[9]Division of Human Genetics, Children's Hospital of Philadelphia, Philadelphia, PA, USA.

[10]Department of Translational and Precision Medicine, University La Sapienza, Rome, Italy.

*devoto@email.chop.edu

Supplementary Figures

Supplementary Figure 1: Principal component analysis of pairwise genetic distances in cases and controls with the 1000 Genome Project Phase 3 samples

Supplementary Figure 2: Density plots of the distribution of variant allele fraction observed in cases

Supplementary Figure 1: Principal component analysis of pairwise genetic distances in case samples

Supplementary Figure 4: Density plot showing the distributions of ratio of transitions to transversions (Ts/Tv)

Supplementary Figure 5: QQ-plot comparing the observed and expected p-values from the case-control burden analysis

Supplementary Figure 6: Pedigree showing the *de novo* variant identified in *STIP1* (A), IGV snapshopt of the read pile-up around the variant (B), and the Sanger trace from the validation experiment (C)

Supplementary Figure 7: Pedigree showing the *de novo* variant identified in *REV1* (A), IGV snapshopt of the read pile-up around the variant (B), and the Sanger trace from the validation experiment (C)

Supplementary Figures

Principal component analysis (PCA) of cases and controls along with population samples from 1000 Genomes project

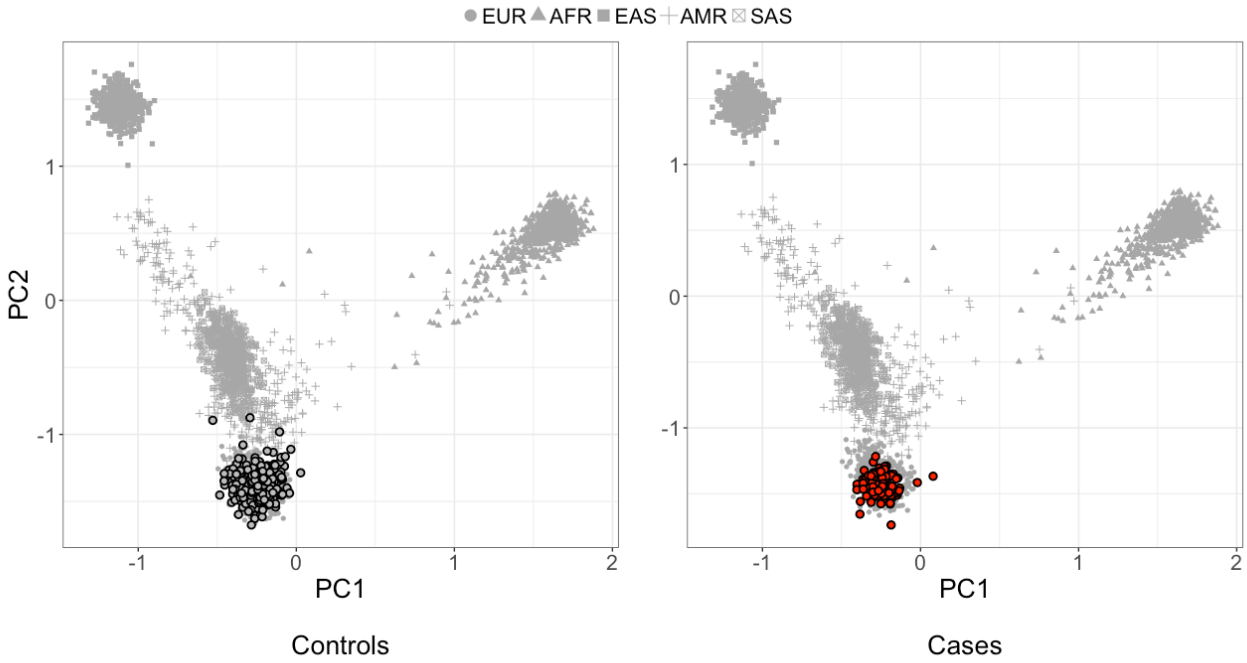

Supplementary Figure 1 Principal component analysis of pairwise genetic distances in cases and controls with the 1000 Genome Project Phase 3 samples

Peddy software tool was used to infer genetic ancestry from the exome sequencing data by comparing our cases and controls against the samples from 1000 Genomes Project phase 3. First plot shows the clustering of controls (gray circles with black outline) and the second plot shows the cases (red circles with black outline). Cases and controls overlap with the European population samples as well as with each others.

Distribution of the variant allele fraction in cases

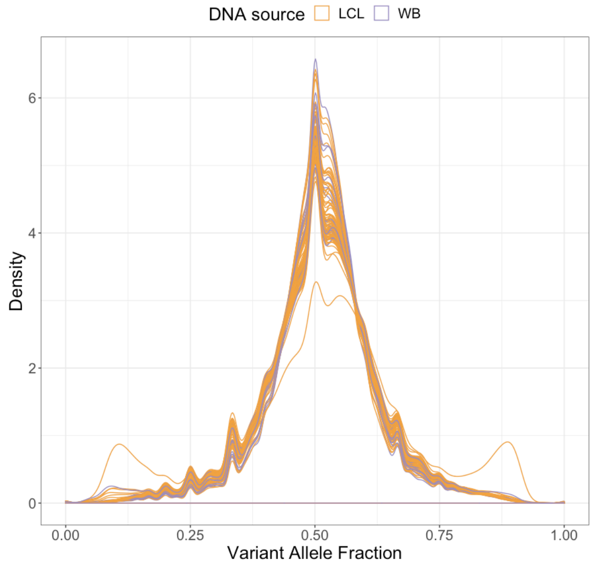

Supplementary Figure 2 Density plot of the distribution of variant allele fraction observed in cases

Variant Allele fraction was calculated using an in-house script by dividing the alternate allele read-depth against the total read-depth at a given location (alt/(ref+alt)). Density plots showed no difference between the samples sequenced from whole-blood (15 WB/ purple lines) or the lymphoblastoid cell lines (85 LCL/ orange lines).

PCA in 100 BA cases stratified by DNA source

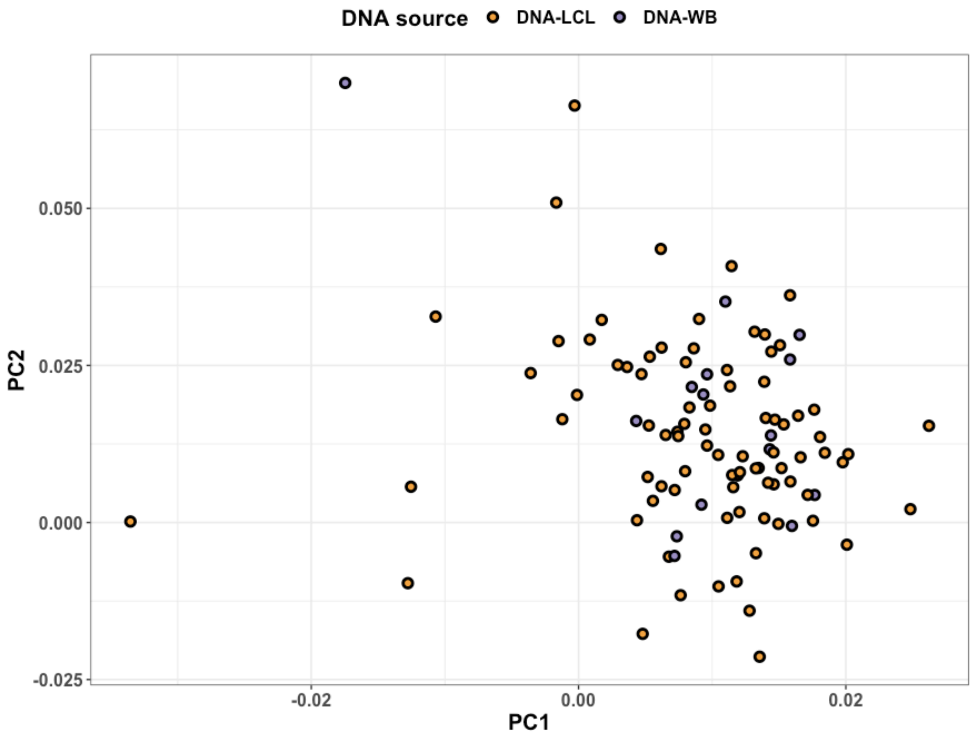

Supplementary Figure 3 Principal component analysis of pairwise genetic distances in case samples

PCA analysis in 100 BA cases shows no systematic differences between the samples sequenced from whole-blood (purple circles) and lymphoblastoid cell-lines (golden circles). PCA was performed using plink2 (--pca command) after pruning the variant callset to remove variants in linkage disequilibrium and variants with minor allele frequency less than 1% (--indep-pairwise 50 5 0.2 --maf 0.01 command).

Transition/ Transversion (Ts/Tv) ratio of 100 BA cases stratified by DNA source

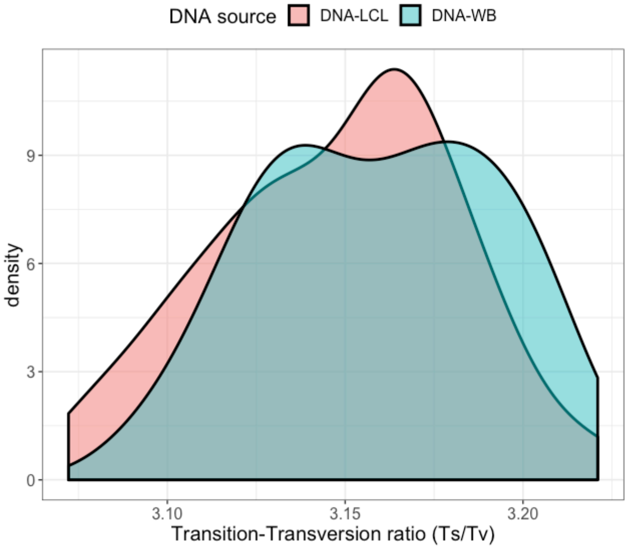

Supplementary Figure 4 Density plot showing the distributions of ratio of transitions to transversions (Ts/Tv)

Distribution of the ratio of transitions to transversions (Ts/Tv) stratified by the DNA-source does not show any difference between the samples sequenced from whole-blood and the lymphoblastoid cell-lines.

QQ-plot of observed p-values from gene-level burden tests using ExactCMC

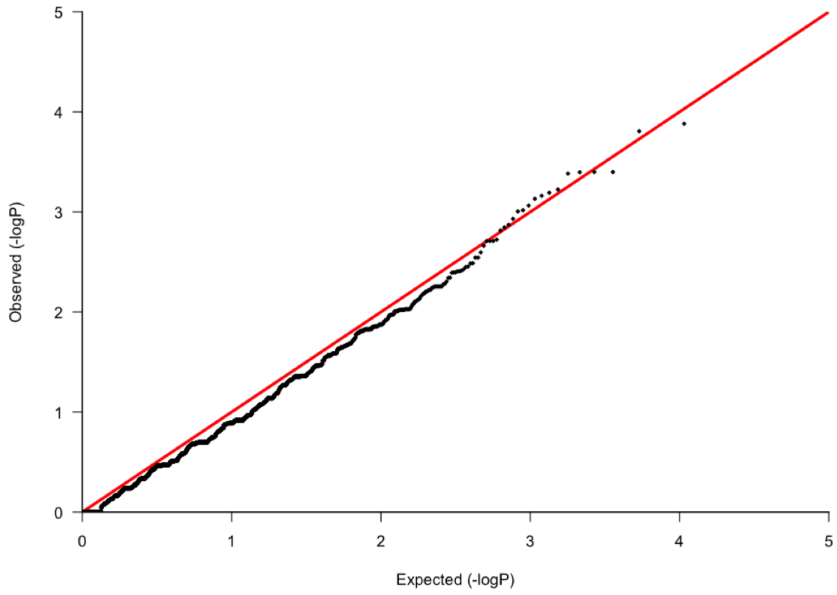

Supplementary Figure 5 QQ-plot comparing the observed and expected P-values from the case-control burden analysis

The observed P-values from the gene-based burden tests were compared to the expected distribution of P-values using a QQ-plot, and only genes with at least three low frequency or rare variants were included.

Sanger traces, and the read pileup of the de novo variants identified in *STIP1* and *REV1*

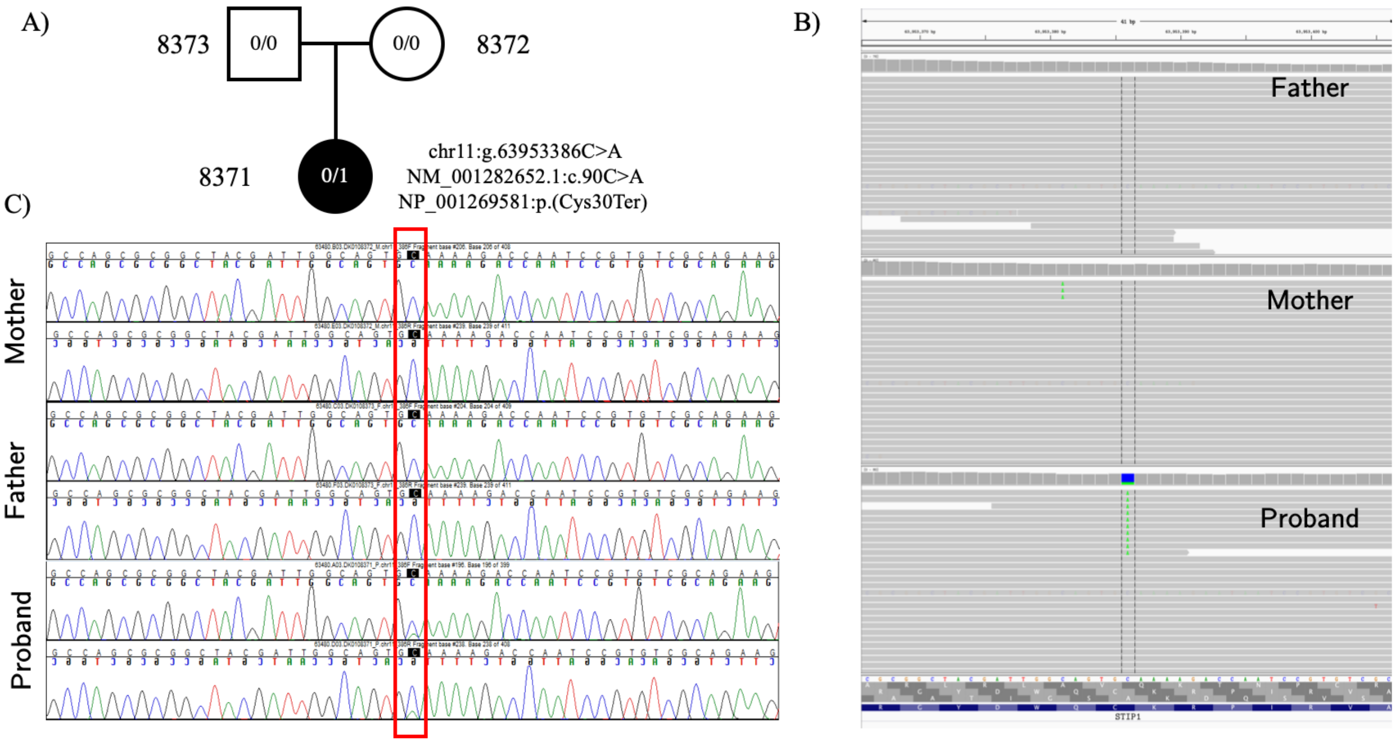

Supplementary Figure 6 Pedigree showing the de novo variant identified in STIP1 (A), IGV snapshot of the read-pileup of the variant (B), and Sanger trace from the validation experiment (C)

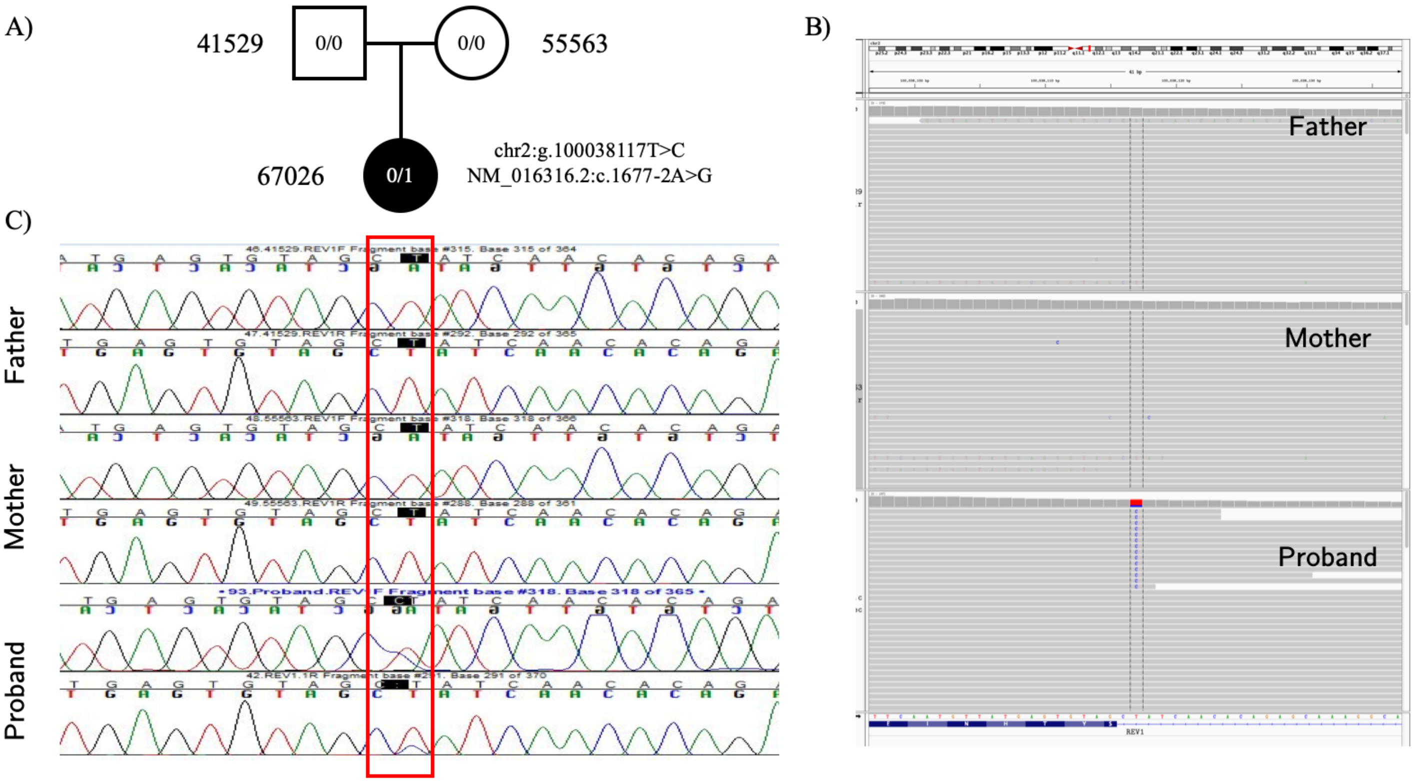

Supplementary Figure 7 Pedigree showing the de novo variant identified in REV1 (A), IGV of the read-pileup of the variant (B), and Sanger trace from the validation experiment (C)
